# Antennal Transcriptome and Differential Expression Analysis of Five Chemosensory Gene Families from the Asian Honeybee *Apis cerana cerana*

**DOI:** 10.1371/journal.pone.0165374

**Published:** 2016-10-24

**Authors:** Huiting Zhao, Yali Du, Pengfei Gao, Shujie Wang, Jianfang Pan, Yusuo Jiang

**Affiliations:** 1 College of Life Science, Shanxi Agricultural University, Taigu, Shanxi, China; 2 College of Animal Science and Technology, Shanxi Agricultural University, Taigu, Shanxi, China; USDA Agricultural Research Service, UNITED STATES

## Abstract

Chemosensory genes play a central role in sensing chemical signals and guiding insect behavior. The Chinese honeybee, *Apis cerana cerana*, is one of the most important insect species in China in terms of resource production, and providing high-quality products for human consumption, and also serves as an important pollinator. Communication and foraging behavior of worker bees is likely linked to a complex chemosensory system. Here, we used transcriptome sequencing on adult *A*. *c*. *cerana* workers of different ages to identify the major chemosensory gene families and the differentially expressed genes(DEGs), and to investigate their expression profiles. A total of 109 candidate chemosensory genes in five gene families were identified from the antennal transcriptome assemblies, including 17 OBPs, 6 CSPs, 74 ORs, 10 IRs, and 2SNMPs, in which nineteen DEGs were screened and their expression values at different developmental stages were determined in silico. No chemosensory transcript was specific to a certain developmental period. Thirteen DEGs were upregulated and 6were downregulated. We created extensive expression profiles in six major body tissues using qRT-PCR and found that most DEGs were exclusively or primarily expressed in antennae. Others were abundantly expressed in the other tissues, such as head, thorax, abdomen, legs, and wings. Interestingly, when a DEG was highly expressed in the thorax, it also had a high level of expression in legs, but showed a lowlevel in antennae. This study explored five chemoreceptor superfamily genes using RNA-Seq coupled with extensive expression profiling of DEGs. Our results provide new insights into the molecular mechanism of odorant detection in the Asian honeybee and also serve as an extensive novel resource for comparing and investigating olfactory functionality in hymenopterans.

## Introduction

Olfactory sensing by the antennae is crucial for insect survival, reproduction, and intraspecific communication. Peripheral odor information is detected by olfactory receptor neurons, most of which are housed in the chemosensillae of the antennae [1]. Diverse olfactory protein families are involved in different steps of peripheral olfactory signal transduction events, such as odorant binding proteins (OBPs), chemosensory proteins (CSPs), odorant receptors (ORs), ionotropic receptors (IRs), and sensory neuron membrane proteins (SNMPs) [2, 3]; however, OR proteins are considered the most important [4].

The OBPs of insects are small and water-soluble and accumulate in the sensillar lymph. They are considered to be the first protein in the olfactory recognition procedure [5]. Odorant molecules are hydrophobic, and are thought to be bound by OBPs and transported through the aqueous sensillar lymph to chemoreceptor proteins located on the surface of olfactory sensory neuron OSN dendrites [6]. Like OBPs, CSPs are small soluble binding proteins and are more conserved than OBPs [7]. CSPs are not only involved in semiochemical detection [8], but may also participate in some non-chemosensory functions [9, 10].

ORs and IRs are two chemosensory receptors families located in the dendritic membrane of OSNs and both are regarded as key factors in the chemosensory signal transduction process [11]. ORs are multi-transmembrane domain receptors and have been shown to function as heteromeric ion channels, consisting of a conventional olfactory receptor and a highly conserved member which is referred to as Orco [12, 13]. IRs appear to be far more ancient than ORs; they are related to the ionotropic glutamate receptors (iGluRs) family but contain a divergent ligand-binding domain.IRs are expressed in coeloconic OSNs and may function in the detection of acids and ammonia [3, 14].

SNMPs are also membrane proteins with two transmembrane helical domains [15]. Insects generally have only one or two genes that encode SNMPs, and their exact functions are mostly unknown [2].

Recently, some of the characteristics of these olfactory-related gene families and their functions have been determined, but the detailed relationships among these proteins, including their synergistic or antagonistic effects in the olfactory recognition process, have received less attention from researchers.

The honeybee is a eusocial insect and key model in the study of olfactory perception and learning behavior [16, 17]. A colony is composed of a single queen, hundreds of male drones, and thousands of sterile female workers. They possess specific social roles: the queen is responsible for laying eggs, drones function in mating with the queen, and workers perform different tasks at different ages, such as taking care of the brood and the queen, building nests, gathering various resources, and guarding the colony [18]. In this society, the sense of chemoreception plays a fundamental role in mediating the wide range of behaviors [19], and honeybee might be expected to have a more complex olfactory system than solitary insects [20].

In the genus *Apis*, the western honeybee (*Apis mellifera* L.) and eastern honeybee (*Apis cerana* Fabr.) are the two most important species. Olfactory related genes was relatively earlier researched in *A*. *mellifera*, such as the OBP genes *ASP1* and *ASP2*, the CSP gene *ASP3* and the OR gene *Or2* were the first to be identified and characterized in Hymenoptera [21–25]. The elucidation of the complete genome sequence of *A*. *mellifera* has facilitated molecular biological research and also resulted in the identification of chemosensory genes [20].To data, 170 ORs, 21 OBPs, 6 CSPs, 10IRs, and 2SNMPs have been annotated [14, 26–29]. Recently, the genome of *A*. *cerana* was sequenced using next-generation technology, and two chemoreceptor families, ORs and IRs, were characterized in the transcriptome data [30].

*Apis cerana cerana* is an economically important species indigenous to China. Compared to *A*. *mellifera*, *A*. *c*. *cerana* possesses unique characteristics such as using sporadic resources of nectar and pollen, good foraging ability, and strong resistance to parasitic mites. As a consequence of these excellent characteristics, there is considerable interest in the olfactory system of the Asian bee. Many olfactory genes have been identified, particularly OBP and CSP genes [31–34]. Previously, we cloned three OR genes and one OBP gene from *A*. *c*. *cerana* and described their expression characteristics [35–37].

Worker bees undertake different tasks in and outside the hive across their life span. Such variation in their behavioral repertoires at different times of the lifecycle is likely regulated by different expression patterns of many genes, including the olfactory genes. In this study, we investigated the antennal chemosensory gene families and compared their expression patterns at different developmental stages in *A*. *c*. *cerana* using an Illumina RNA-Seq approach. Overall, 109 chemosensory genes were identified including 17 OBPs, 6 CSPs, 74 ORs, 10 IRs, and 2 SNMPs. Differentially expressed genes (DEGs) were screened and their spatio-temporal expression profiles were analyzed in combination with quantitative real-time PCR. The aim of this study was to explore the relationship between chemosensory genes and the division of labor of worker bees. The results clearly showed an age-biased expression of some chemosensory genes and ultimately allowed us to identify potential targets in production management of the colony. The data also provide a valuable resource for further genetic and molecular studies on the olfactory recognition mechanism of honeybees.

## Materials and Methods

### Insects and tissue samples

Honeybees (*A*. *c*. *cerana*) were sampled from March to July from an apiary of Shanxi Agricultural University, China. Frames with bee pupae close to eclosion were taken from two hives and incubated in an environmental chamber at a constant temperature of 33°C and80% humidity. After eclosion, the workers were marked and returned to their hives until sampling. For transcriptome sequencing, the antennae of about 200 worker bees from two hives at 1, 10, 15, and 25 days of age were dissected, respectively. The major body parts (antenna, head, thorax, abdomen, legs, and wings) of 10-day-old worker bees were separated for qPCR analysis.

### RNA-Seq library construction and sequencing

Samples were collected, and immediately frozen in liquid nitrogen and then stored at -80°C until extraction. Total RNA was isolated with TRIzol reagent (Invitrogen, Carlsbad, CA, USA) according to the manufacturer’s instructions. Residual DNA in the total RNA was removed using DNase (Promega, Madison, WI, USA). First-strand cDNA was synthesized using reverse transcriptase (Invitrogen, Carlsbad, CA, USA). Eight cDNA libraries from four developmental stages were processed for RNA-Seq analysis independently using the reagents provided in the Illumina sequencing kit according to the manufacturer’s instructions. Finally, the eight cDNA libraries were sequenced using the Illumina HiSeq 2500 platform at Biomarker Technologies Co., Ltd (Beijing, China).

### Assembly

The raw reads were cleaned by removing the adapter sequences and low-quality sequences (reads with ambiguous bases ‘N’, and reads with more than 10% Q <20 bases).Clean reads were then mapped back to contigs using Trinity software, with the parameters set at a similarity of 90% [38]. Next, by performing pair-end joining and gap filling, contigs were assembled into transcripts and were subsequently clustered to obtain unigenes. In this study, all Illumina sequencing data was submitted to the SRA of NCBI (accession number:SRR3180625).

### Gene annotation and chemosensory gene identification

Unigenes longer than 200 bp were first aligned by BLASTX against the non-redundant protein database, including Nr, Swiss-Prot, KEGG, GO, and COG at a threshold E-value of 10^-5^.Functional annotation was performed by retrieving proteins with the highest sequence similarity with the given unigene. Then, GO terms were assigned using the Blast2GO algorithm [39]. The transcripts were categorized as cellular component, molecular function, or biological process, allowing for a general quality assessment.

To identify the putative chemosensory genes of *A*. *c*. *cerana*, sequences whose annotations corresponded best to OBPs, CSPs, ORs, IRs, or SNMPs were retained as candidate genes. Next, these genes were manually revised by further analysis using the BLASTN algorithm in the non-redundant nucleotide database acquired at NCBI. ORs were further verified in a custom-made database of known *A*. *mellifera* query protein sequences [29].

The ORFs of the candidate chemosensory genes were determined using the ORF Finder tool (http://www.ncbi.nlm.nih.gov/gorf/gorf.html). Putative N-terminal signal peptides of candidate OBPs and CSPs were predicted by the program Signal IP 4.0 (http://www.cbs.dtu.dk/services/SignalP/) [40]. Transmembrane domains of novel candidate ORs, IRs, and SNMPs were determined using TMHMM server v2.0 [41]. Phylogenetic trees were constructed based on the amino acid sequences of putative *A*. *c*. *cerana* chemosensory genes and homologous sequences reported in other hymenopteran species. Amino acid sequences were aligned using Clustal W, then unrooted neighbor-joining trees were constructed using MEGA6.0 [42] and the branch support was assessed with 1000 bootstrap replications. Dendrograms were viewed and graphically edited in FigTree v1.4.2 (http://tree.bio.ed.ac.uk/software/figtree/).

### Identification of chemosensory DEGs and gene expression analysis

To compare gene expression levels between different libraries, the expression abundance of all unigenes were calculated by the RPKM (reads per kilobase of transcript per million mapped reads) algorithm [43]. DEGs in the 8 libraries were identified via a rigorous statistical algorithm based on Audic and Claverie’s method [44]. A threshold for FDR (false discovery rate) of <0.01and an absolute expression value of log_2_FC (fold change) ≥1 were used to determine significant differences in gene expression. As a result, we acquired all DEGs related to olfaction and their RPKM values.

To verify the expression values of the chemosensory DEGs identified from our transcriptome and to further investigate their tissue expression profiles, qPCR was performed. Specific primers (S1 Table) were designed using primer3plus (http://www.bioinformatics.nl/cgibin/primer3plus/primer3plus.cgi). qPCR was performed using aSYBR^®^Premix Ex Taq^™^ Kit(Takara, Dalian, China), which was run on a Mx3000PqPCRSystem (Stratagene, La Jolla, CA,USA). The cycling conditions were as follows: denaturation at 95°C for 20 s, followed by 40 cycles of 95°C for 15 s, and 60°C for 20 s. A melting curve analysis was then performed at 95°C for 20 s, 60°C for 30 s, and 95°C for 30 s in order to judge the specificity of the PCR products.

Three biological replicates were performed for each tested gene, and three technical replicates were performed for each biological replicate. Negative controls were non-template reactions (replacing cDNA with ddH_2_O). Relative quantification was analyzed relative to the control gene *Arp1* using the comparative 2^-ΔΔCt^ method [45].

## Results and Discussion

### Summary of transcriptome sequencing data

Illumina HiSeq RNA-Seq technology has become popular over the last decade because of its advantages of higher throughput and lower cost. This approach has been used in an increasing number of studies of different insect species, to identify total genes expressed in specific cells or tissues; determine relative expression abundance; discover SNP sites at the transcriptional level in different transcripts and alternative splice sites of specific genes; reveal genetic polymorphism and genetic markers (SSRs); and detect unknown genes and new transcripts.

In this study, data on the antennal transcriptome of *A*. *c*. *cerana* was generated using Illumina HiSeq 2500 technology. After filtering the raw reads, 8 high-quality unigene libraries were established, composed of 184.04M clean reads, and in each library the Q30>90% (see S2 Table). These clean reads were assembled into 4,235,071 contigs. After merging and clustering, 125,072 unigenes were acquired with a mean length of 760 nt and an N50 length of 1,151 nt. Approximately 43% of these unigenes (53,576) were more than 500nt in length (see S3 Table). Each unigene was given a unique gene ID.

### Gene ontology annotation

**Fig 1:**
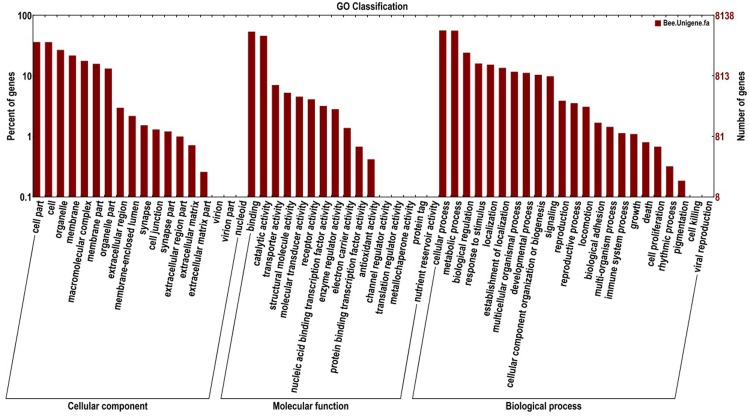
Functional annotation of assembled unigenes based on gene ontology (GO) categorization. The unigenes were assigned into three main categories: cellular component, molecular function, and biological process. The three main categories were categorized further to 47 subcategories using the Blast2GO program.

Through annotation by BLASTX in 5 protein databases, 16,762 unigenes were matched to known proteins. GO annotation was used to classify the transcripts into functional groups according to GO category. One transcript could align to more than one biological process; therefore, a total of 34,051 unigenes were assigned into three main GO terms (Fig 1): 14,575 unigenes were aligned in the cellular component category, 10,541 in the molecular function category, and 19,757 in the biological process category. In these categories, some subcategories had high percentages, such as cell (20.21%) and cell part (20.25%) in the cellular component category, binding (41.61%) and catalytic activity (35.56%) in the molecular function category, and cellular process (23.24%) and metabolic process (23.07%) in the biological process category, suggesting that transcripts in the antennae of *A*. *c*. *cerana* cover a wide spectrum of biological processes.

### Putative chemosensory gene families and their expression profiles

The molecular basis of chemoreception in the Asian honeybee is not as well understood as that of its sibling species, the Western honeybee (*A*. *mellifera*). In this study, we primarily focused on five crucial repertoires of chemosensory genes (OBPs, CSPs, ORs, IRs, and SNMPs). Candidate genes were identified by keyword searches and manually analyzing the annotated unigenes. In addition, we also constructed a local nucleotide sequence database of the RNA-Seq unigenes, in which ORs and IRs were also queried based on similar sequences of *A*. *mellifera* [14, 29]. Using this method, we identified 109 chemosensory genes in the antennal transcriptome of *A*. *c*. *cerana*, including 17 OBPs, 6 CSPs, 74 ORs, 10 IRs, and 2SNMPs. These candidate chemosensory unigenes were named by a four-letter species abbreviation in accordance with established conventions. Gene names were preferentially given the same name when they were orthologous to a sequence in *A*. *mellifera* (e.g.,*AcerOBP1*, *AcerIR8a*). Of these transcripts, 91 unigenes had full length ORFs. Sequence accuracy was verified by conventional PCR and sequencing on 10 selected unigenes (data not shown). In phylogenetic trees, almost all candidate chemosensory genes clustered with at least one *A*. *mellifera* (S1–S5 Figs).In the olfactory receptor gene families, we identified the coreceptor gene *OR2* in the OR family and *IR25a* and *IR8a* in the IR family cluster along with the respective orthologs of other hymenopteran species. In the OBP family dendrogram, members of the Minus-C class of *A*. *c*. *cerana* and *A*. *mellifera* were clustered into a clade, with their second and fifth cysteine residues missing. Other information tied to these unigenes, including the gene name, amino acid sequence length, transmembrane domains, signal peptide, and sequence similarity are listed in Tables 1–5.

**Table 1:** Candidate OBPs identified in *A*. *c*. *cerana* antennae.

Gene ID	Gene Name	Complete ORF	ORF (aa)	Signal peptide	RPKM value								Ortholog gene of *A*. *mellifera*		
					T1-1	T1-2	T2-1	T2-2	T3-1	T3-2	T4-1	T4-2	Gene^a^	E-Value	Identity(%)
c42411.graph_c2	OBP1	Yes	143	1–24	11297.52	10172.42	17575.82	22798.16	15741.55	15675.90	16879.60	19211.18	OBP1	2.00E-92	89
c45966.graph_c0	OBP2	Yes	142	1–19	10480.13	9871.22	10697.94	14529.16	9295.87	9686.50	11250.54	12430.88	OBP2	6.00E-96	94
c65620.graph_c0	OBP3	Yes	140	1–22	1.47	1.90	1.70	1.80	2.01	8.42	1.14	1.13	OBP3	8.00E-84	91
c54406.graph_c0	OBP4	Yes	137	1–20	215.63	224.11	312.39	360.66	285.40	316.95	338.04	343.46	OBP4	2.00E-74	78
c68504.graph_c0	OBP5	Yes	146	1–26	1534.36	1514.49	2684.39	3403.12	2221.80	2314.48	2647.13	2847.31	OBP5	3.00E-95	89
c68504.graph_c0	OBP6	Yes	146	1–24	1534.36	1514.49	2684.39	3403.12	2221.80	2314.48	2647.13	2847.31	OBP6	1.00E-87	90
c54600.graph_c0	OBP7*	Yes	145	1–19	8.01	7.76	42.99	47.09	44.67	43.33	57.24	59.82	OBP7	3.00E-80	86
c29112.graph_c0	OBP9	Yes	132	1–23	0.23	0.27	0.69	0.46	0.82	0.13	0.57	0.46	OBP9	6.00E-93	98
c60858.graph_c0	OBP10	Yes	149	1–22	60.37	54.31	23.13	20.25	19.81	17.88	15.35	18.77	OBP10	7.00E-858	96
c66453.graph_c0	OBP11	Yes	142	NO	46.49	48.33	69.35	75.59	57.95	62.45	67.16	69.41	OBP11	2.00E-88	90
c54514.graph_c0	OBP12*	Yes	150	1–22	17.89	19.80	42.92	45.59	35.78	39.47	36.83	36.72	OBP12	3.00E-85	82
c26273.graph_c0	OBP13*	Yes	132	1–17	10.17	11.18	0.28	0.47	0.50	0.14	0.86	0.31	OBP13	2.00E-87	98
c25191.graph_c1	OBP14*	Yes	132	1–23	181.60	185.99	524.50	503.91	484.50	503.21	572.24	576.40	OBP14	1.00E-67	85
c42419.graph_c0	OBP15*	Yes	135	1–16	129.57	140.81	1026.38	1177.24	906.41	1031.21	853.18	837.03	OBP15	5.00E-66	69
c20845.graph_c0	OBP17*	Yes	135	1–16	8.39	12.48	1.68	2.06	1.00	1.21	0.87	2.30	OBP17	6.00E-23	100
c42400.graph_c0	OBP19	Yes	139	1–16	806.12	826.98	831.49	899.51	585.67	679.84	625.91	571.75	OBP19	1.00E-54	65
c69395.graph_c5	OBP21*	Yes	135	1–16	1788.66	1797.92	3820.02	4179.71	3064.26	3380.04	4070.38	3914.71	OBP21	7.00E-73	81

"*" represent the DEGs. Gene^a^ indicate genes identified by Forêt and Maleszka [26].

T1-1 and T1-2 are biological replicates of 1-day-old worker bees; T2-1and T2-2 are biological replicates of 10-day-old worker bees; T3-1andT3-2 are biological replicates of 15-day-old worker bees;T4-1andT4-2 are biological replicates of 25-day-old worker bees (these definitions apply to the other Tables).

**Table 2:** Candidate CSPs identified in *A*. *c*. *cerana* antennae.

Gene ID	Gene Name	Complete ORF	ORF (aa)	Signal peptide	RPKM value								Ortholog gene of *A*. *mellifera*		
					T1-1	T1-2	T2-1	T2-2	T3-1	T3-2	T4-1	T4-2	Gene^b^	E-Value	Identity(%)
c71123.graph_c0	CSP1	Yes	116	1–19	2555.66	2621.70	2329.78	2475.67	2096.84	2224.65	2449.57	2452.29	CSP1	6.00E-79	94
c59619.graph_c0	CSP2*	Yes	117	1–21	168.15	145.54	51.43	61.68	37.30	38.30	32.51	34.78	CSP2	5.00E-81	98
c71125.graph_c0	CSP3	Yes	131	1–19	4525.12	4442.88	2566.17	2992.54	2660.61	2526.92	2809.55	2774.02	CSP3	1.00E-82	97
c68379.graph_c8	CSP4	Yes	128	1–20	221.01	215.14	200.47	230.71	174.02	176.66	180.37	190.16	CSP4	3.00E-85	94
c71983.graph_c0	CSP5*	Yes	104	1–19	29.08	24.13	3.40	5.18	4.70	5.17	5.11	3.88	CSP5	8.00E-68	97
c15320.graph_c0	CSP6*	Yes	125	1–18	34.92	40.04	6.18	6.53	3.77	3.05	3.47	2.87	CSP6	6.00E-88	98

"*" represent the DEGs. Gene^b^ indicate genes identified by Forêt *et al*.[27].

**Table 3:** Candidate ORs identified in *A*. *c*. *cerana* antennae.

Gene ID	Gene Name	Complete ORF	Orf (aa)	Tmd (No.)	Rpkm value								Ortholog gene of *A*. *mellifera*		
					T1-1	T1-2	T2-1	T2-2	T3-1	T3-2	T4-1	T4-2	Gene^c^	E-Value	Identity(%)
c69500.graph_c0	OR1	Yes	400	6	18.11	17.93	21.07	15.44	18.34	21.68	21.33	22.01	OR1	0	98
c67239.graph_c0	OR2	Yes	478	7	177.58	179.83	188.42	181.76	176.40	194.40	181.56	196.76	OR2	0	99
c70127.graph_c0	OR5	Yes	401	6	35.19	37.93	48.50	37.72	49.66	48.57	52.37	50.15	OR5	0	94
c68791.graph_c0	OR13	No	–	–	43.04	42.09	60.93	62.25	55.46	64.06	63.00	68.67	OR13	0	97
c70257.graph_c0	OR16	No	–	–	73.42	73.70	86.33	80.24	72.33	81.32	85.89	88.09	OR16	1.00E-161	95
c70257.graph_c0	OR18	Yes	411	6	73.42	73.70	86.33	80.24	72.33	81.32	85.89	88.09	OR18	0	98
c66900.graph_c0	OR20	No	–	–	13.52	13.63	15.33	15.03	15.01	16.11	18.59	17.78	OR20	0	96
c66900.graph_c0	OR23	No	–	–	13.52	13.63	15.33	15.03	15.01	16.11	18.59	17.78	OR23	4.00E-161	86
c68828.graph_c1	OR24	No	–	–	22.40	22.01	29.31	27.26	25.54	27.50	29.65	31.37	OR24	1.00E-137	95
c67366.graph_c0	OR26	Yes	403	5	20.76	22.02	32.38	32.74	24.96	31.66	32.81	30.43	OR26	0	93
c67366.graph_c0	OR27	Yes	405	8	20.37	21.56	31.82	32.24	24.39	31.14	32.15	29.85	OR27	0	97
c65220.graph_c0	OR28*	Yes	405	6	3.68	3.92	7.55	7.14	7.75	8.00	7.20	7.19	OR28	0	96
c70242.graph_c0	OR35	Yes	413	7	10.85	10.73	14.39	12.69	14.81	16.05	16.18	16.57	OR35	0	95
c69441.graph_c0	OR46	Yes	406	4	63.52	72.85	62.47	62.59	49.91	59.54	61.58	63.72	OR46	0	92
c68433.graph_c0	OR53	Yes	409	5	42.72	44.52	40.93	34.44	38.73	45.21	42.57	44.99	OR53	0	89
c69344.graph_c0	OR57	Yes	407	6	17.32	17.56	21.85	18.67	23.75	25.43	26.07	27.63	OR57	0	97
c68433.graph_c0	OR58	No	–	–	42.72	44.52	40.93	34.44	38.73	45.21	42.57	44.99	OR58	1.00E-170	98
c65302.graph_c0	OR62	Yes	385	5	7.50	6.26	7.40	6.83	6.02	6.53	5.22	6.00	OR62	0	88
c69134.graph_c0	OR64	Yes	393	6	21.92	21.45	32.82	37.90	30.63	33.40	36.22	36.70	OR64	0	94
c68872.graph_c0	OR68	Yes	375	6	3.46	3.83	3.18	3.23	3.48	4.25	4.20	4.13	OR68	0	100
c56937.graph_c0	OR70	Yes	372	5	13.26	13.44	16.77	20.48	17.49	17.84	18.95	19.48	OR70	0	98
c69447.graph_c0	OR71	Yes	371	5	12.05	13.67	17.09	20.10	15.18	17.22	16.97	17.92	OR71	0	98
c69447.graph_c0	OR72	Yes	370	6	12.05	13.67	17.09	20.10	15.18	17.22	16.97	17.92	OR72	0	95
c67615.graph_c0	OR73	Yes	388	3	3.10	2.75	3.15	2.68	2.81	3.20	2.29	3.22	OR73	0	90
c69036.graph_c4	OR74	Yes	402	6	13.81	11.93	12.76	14.29	14.93	15.62	14.65	14.23	OR74	0	91
c70270.graph_c0	OR76	Yes	426	6	4.23	3.91	4.95	6.25	4.77	4.41	5.95	7.09	OR76	0	86
c65858.graph_c0	OR81	Yes	405	6	19.29	17.43	19.09	25.78	19.14	22.11	23.17	23.02	OR81	0	92
c69036.graph_c4	OR84	Yes	392	5	13.56	11.68	12.54	14.07	14.59	15.36	14.36	13.96	OR84	0	82
c68298.graph_c3	OR85	Yes	411	8	5.73	5.76	6.88	6.78	5.69	6.46	7.88	8.14	OR85	0	93
c68298.graph_c3	OR86	Yes	403	5	5.73	5.76	6.88	6.78	5.69	6.46	7.88	8.14	OR86	0	92
c65378.graph_c0	OR87	Yes	414	7	3.91	4.09	4.67	3.96	4.76	4.95	4.55	4.80	OR87	0	97
c70217.graph_c0	OR88	Yes	431	7	15.56	15.46	19.06	23.93	19.18	21.60	20.89	21.65	OR88	0	90
c70217.graph_c0	OR90	Yes	407	7	15.56	15.46	19.06	23.93	19.18	21.60	20.89	21.65	OR90	0	93
c69881.graph_c0	OR91	No	404	–	24.40	27.29	33.51	34.98	30.79	33.91	33.22	38.43	OR91	0	87
c69406.graph_c0	OR94	Yes	407	6	8.17	8.09	11.41	11.05	10.65	12.20	11.41	11.70	OR94	0	91
c69189.graph_c0	OR96	Yes	410	7	3.38	4.16	3.62	3.48	3.63	4.71	4.34	5.31	OR96	0	96
c56936.graph_c0	OR97	Yes	395	6	1.08	1.04	0.93	0.69	1.67	2.33	1.45	1.59	OR97	0	94
c68538.graph_c0	OR100	Yes	414	4	2.46	3.36	3.61	3.15	2.89	2.74	3.40	4.37	OR100	0	90
c70178.graph_c0	OR101	Yes	406	3	12.08	13.11	14.43	12.07	13.27	15.08	14.96	15.16	OR101	0	95
c69146.graph_c0	OR103	Yes	403	4	32.79	31.60	30.42	23.18	29.91	32.34	36.23	36.82	OR103	0	95
c69773.graph_c0	OR104	Yes	405	4	11.52	10.92	10.75	7.08	12.40	11.83	14.17	13.29	OR104	0	96
c70529.graph_c0	OR106	Yes	388	7	16.01	15.53	17.06	17.73	17.62	19.37	21.47	21.31	OR106	0	91
c70529.graph_c0	OR107	Yes	391	5	16.01	15.53	17.06	17.73	17.62	19.37	21.47	21.31	OR107	0	95
c65323.graph_c0	OR109	Yes	392	6	29.29	29.74	33.98	34.83	27.77	32.23	36.28	37.44	OR109	0	89
c70236.graph_c0	OR112	Yes	388	6	4.19	3.69	3.24	3.25	3.74	3.97	3.92	4.70	OR112	0	96
c67559.graph_c1	OR113*	Yes	389	6	1.87	2.21	4.49	4.17	3.71	3.41	4.83	3.90	OR113	0	94
c66726.graph_c0	OR114	Yes	392	6	5.60	3.95	4.27	3.64	6.42	5.24	4.80	5.01	OR114	0	93
c64278.graph_c0	OR115	Yes	399	6	10.83	8.17	16.94	18.85	15.61	16.69	16.65	17.29	OR115	0	95
c72753.graph_c0	OR116	No	206	–	2.22	2.56	3.41	2.12	3.30	3.48	2.76	3.04	OR116	9.00E-106	96
c36301.graph_c0	OR117	No	271	–	1.33	1.05	1.82	1.93	1.04	0.84	1.73	1.83	OR117	1.00E-149	93
c28430.graph_c1	OR118	No	–	–	1.02	1.02	0.99	0.73	0.39	1.02	1.27	0.99	OR118	2.00E-171	96
c66411.graph_c0	OR119*	Yes	411	7	0.59	0.62	1.05	0.79	1.38	1.37	0.74	1.12	OR119	0	86
c61779.graph_c0	OR120	No	–	–	4.72	4.66	4.67	2.64	3.73	4.27	3.93	4.30	OR120	1.00E-122	96
c53036.graph_c0	OR124	No	–	–	0.74	0.43	0.90	0.74	0.53	1.29	0.23	0.25	OR124	7.00E-58	78
c71095.graph_c0	OR125	No	–	–	7.69	7.71	7.16	4.63	5.20	6.19	6.68	9.00	OR125	8.00E-64	67
c64197.graph_c1	OR130	No	–	–	1.94	2.39	2.33	1.72	2.31	2.01	2.01	1.70	OR130	1.00E-128	80
c70677.graph_c0	OR139*	Yes	387	7	8.14	8.50	20.57	20.63	36.20	30.80	19.63	20.14	OR139	2.00E-164	64
c57617.graph_c0	OR140	Yes	428	6	10.10	10.85	10.70	7.87	9.24	10.32	10.82	10.34	OR140	0	95
c70711.graph_c3	OR141*	Yes	432	7	5.17	4.60	11.67	12.88	9.71	12.38	11.94	12.63	OR141	0	95
c39732.graph_c0	OR142	Yes	375	6	0.56	0.45	0.73	0.57	0.55	0.60	0.53	0.51	OR142	0	94
c68527.graph_c0	OR144	Yes	372	6	12.66	12.92	14.14	12.66	13.01	15.05	15.54	16.53	OR144	0	91
c69284.graph_c0	OR146	Yes	374	8	6.73	5.84	5.53	4.96	6.04	5.75	6.48	6.50	OR146	0	92
c68527.graph_c0	OR151	Yes	366	6	12.43	12.65	13.89	12.46	12.71	14.80	15.22	16.21	OR151	1.00E-120	59
c58084.graph_c0	OR154	Yes	374	6	4.66	5.48	7.52	5.52	5.10	5.68	5.73	5.66	OR154	0	96
c78216.graph_c0	OR156	Yes	364	7	0.94	0.81	0.74	0.25	0.40	0.54	0.59	0.81	OR156	0	95
c57540.graph_c0	OR157	Yes	370	6	0.62	0.49	0.97	1.14	0.95	1.26	1.42	1.32	OR157	0	91
c62462.graph_c0	OR159	Yes	321	5	7.79	7.59	6.02	4.01	8.06	8.31	7.33	6.54	OR159	6.00E-71	76
c67664.graph_c1	OR160	Yes	390	7	13.79	13.40	17.42	16.04	18.83	18.78	19.02	19.10	OR160	0	99
c64215.graph_c0	OR161	Yes	385	5	13.08	12.69	13.75	14.09	11.68	13.72	12.59	15.59	OR161	0	96
c69738.graph_c0	OR162	Yes	410	5	8.34	7.07	11.91	11.70	12.22	11.54	10.00	11.98	OR162	4E-69	91
c66911.graph_c0	OR163	Yes	387	6	6.31	6.93	6.02	6.12	7.53	7.04	7.78	7.20	OR163	0	89
c55975.graph_c0	OR164	Yes	383	5	3.10	3.59	3.41	2.78	2.84	3.85	4.68	3.69	OR164	0	97
c59293.graph_c0	OR167*	Yes	436	5	9.38	10.89	18.55	18.39	17.42	19.48	21.48	21.04	OR167	0	92
c69502.graph_c0	OR170	Yes	397	6	7.68	6.08	6.33	6.25	5.35	5.61	6.10	6.47	OR170	0	89

"*" represent the DEGs. "–" indicate gene that is partial in sequence without intact ORF. TMD − transmembrane domains. Gene^c^ indicate genes identified by Robertson and Wanner [29].

**Table 4:** Candidate IRs identified in *A*. *c*. *cerana* antennae.

Gene ID	Gene Name	Complete ORF	ORF (aa)	TMD (No.)	RPKM value								Ortholog gene of *A*. *mellifera*		
					T1-1	T1-2	T2-1	T2-2	T3-1	T3-2	T4-1	T4-2	Gene^d^	E-Value	Identity(%)
c68056.graph_c0	IR8a	Yes	891	5	16.37	17.14	18.54	18.81	12.28	15.59	14.43	17.22	IR8a	0	93
c67654.graph_c1	IR25a	Yes	703	2	11.14	9.38	9.92	8.29	10.45	9.58	9.82	8.50	IR25a	0	96
c65841.graph_c0	IR68a	NO	–	–	4.22	4.22	3.74	1.95	3.69	3.29	3.42	3.86	IR68a	0	98
c69783.graph_c5	IR75f.1	Yes	–	4	10.92	10.12	9.32	7.28	7.98	8.63	7.27	7.72	IR75f.1	0	97
c53855.graph_c0	IR75f.2	Yes	634	2	1.23	1.07	1.64	1.60	1.60	1.94	1.52	1.72	IR75f.2	0	94
c48736.graph_c0	IR75f.3	NO	–	–	0.84	0.91	1.37	0.59	1.13	0.97	0.80	0.78	IR75f.3	0	90
c67646.graph_c7	IR75u	NO	–	–	4.43	5.94	7.10	6.79	8.45	10.86	1.81	5.24	IR75u	6E-87	92
c53468.graph_c1	IR76b*	Yes	544	5	19.95	19.79	45.92	40.84	30.48	32.20	34.47	37.49	IR76b	0	89
c67321.graph_c2	IR93a	NO	–	–	3.10	3.24	2.33	1.91	2.37	2.46	2.67	2.42	IR93a	0	97
c69866.graph_c3	IR218*	Yes	470	4	289.82	270.02	102.18	101.94	108.45	114.31	93.97	96.62	IR218	0	93

"*" represent the DEGs. "–" indicate gene that is partial in sequence without intact ORF. TMD − transmembrane domains. Gene^d^ indicate genes identified by Croset *et al*.[14].

**Table 5:** Candidate SNMPs identified in *A*. *c*. *cerana* antennae.

Gene ID	Gene Name	Comlplete ORF	ORF (aa)	TMD (No.)	RPKM value								Ortholog gene of *A*. *mellifera*		
					T1-1	T1-2	T2-1	T2-2	T3-1	T3-2	T4-1	T4-2	Gene^e^	E-Value	Identity(%)
c70015.graph_c0	SNMP1	Yes	574	2	194.91	185.96	251.49	262.33	219.02	238.27	244.72	263.95	SNMP1	0	98
c63242.graph_c0	SNMP2*	Yes	511	2	3.46	3.02	5.96	4.82	3.91	4.47	6.52	5.80	SNMPX	0	94

"*" represent the DEGs. TMD − transmembrane domains. Gene^e^ indicate genes identified by Nichols and Vogt [28]

Expression values of the 109 candidate chemosensory unigenes in the 8 different libraries were calculated using the RPKM metric, and their RPKM values are shown in Tables 1–5. The expression correlation between repetitive samples is the most important indicator for rationality and reliability of the RNA-Seq results. In general, the correlation value should be greater than 0.92 (R^2^≧0.92). In our study, the scatter plot showed that the R^2^ between each group of two biological replicates was more than 0.98 (see S4 Table). The RPKM value analysis showed that nearly all of the chemosensory genes were expressed in each library and no unique genes belonged to any library, showing no age bias in chemosensory gene expression. Among the 109 putative genes, 5 OBP genes (*OBP1*, *OBP2*, *OBP5*, *OBP6*, and *OBP21*) and 2 CSP genes(*CSP1* and*CSP3*)were highly expressed in the worker antennal transcriptomes (RPKM values>1000);*OBP1* had the highest expression levels. At the same time, we found that the other three gene families (ORs, IRs and SNMPs)exhibited relatively low expression levels across the whole developmental stages (RPKM values<100), except those of *OR2*, *IR218*, and *SNMP1*.These results were similar to the transcriptome data of *Agrotis ipsilon* [46]. This observation suggested that these antennae-enriched OBP and CSP proteins might play essential roles in olfactory sensory processes for worker bees and that the insects might use a large number of odor binding proteins to monitor these small molecules.

### Homology analysis with the sibling species *A*. *mellifera*

*A*. *mellifera* was the first hymenopteran species to have its genome sequenced, and several chemosensory protein families were found and reported. In this study, the same number of CSPs, IRs and SNMPs were identified in *A*. *c*. *cerana* as in *A*. *mellifera*, whereas the number of ORs and OBPs were relatively fewer. By repeated comparison and analysis, it was found that the missing genes were not due to the lack of expression in the antennae of *A*. *c*. *cerana*, but to the limitation of denovo RNA-Seq. Since some superfamilies have undergone duplications or alternative splicing during the evolutionary process, this may have produced many genes highly homologous to each other, including the ORs. However, the unigenes with high homology were merged and clustered to single sequence during the de novo assembly process. Thus, the number of annotated genes will be less than the actual number. This problem occurs prominently in gene superfamilies.

In order to investigate the conservatism of these putative chemosensory genes, sequence similarity analysis was performed with orthologs from *A*. *mellifera*. In summary, OR family genes were highly divergent, with sequence identities varying from 59% to 100% (Table 3), which is common for insect olfactory receptor genes [12, 47, 48]. In addition, the OBP family also displayed high sequence variation with sequence identities varying from 65% to 98% (Table 1).*OBP9* and *OBP13* shared the highest identity of with their homologs*AmelOBP9* and *AmelOBP13*, whereas*OBP19* showed a low level of sequence similarity with *AmelOBP19*.

Compared with ORs and OBPs, the other two olfactory gene families (IRs and CSPs) were more highly conserved in insects, and their number was generally consistent [7, 14]. In our study, we identified 10 putative IRs and 6 CSPs that shared greater than 90% sequence identity compared to the orthologs of *A*. *mellifera* (see Tables 2 and 4).

SNMPs are another chemosensory protein family that is conserved throughout holometabolous insects [28]. In our study, two SNMP genes were also highly similar to the orthologs of *A*. *mellifera* (>94%) (see Table 5).

### Chemosensory DEGs and their expression profiles

**Fig 2:**
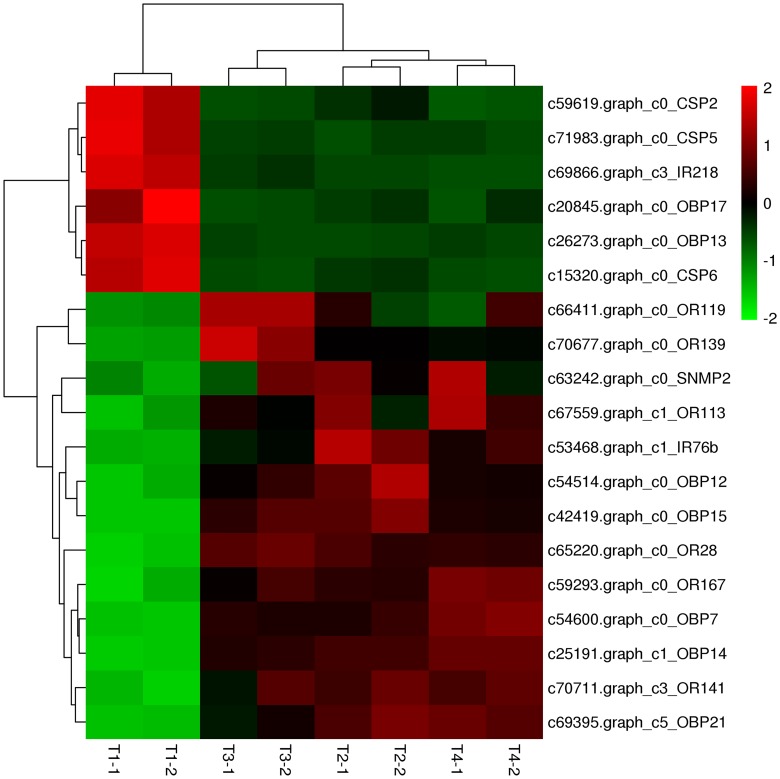
Heatmap of DEGs based on transcriptome data. Clustering of genes is shown on the left of the figure, and the transcript ID and gene name are shown on the right. The color from red to green indicates high to low expression levels.

After estimating gene expression levels, we performed a DEG analysis between two of the eight samples. Using the Audic and Claverie method [44], a total of 1,052 DEGs were detected, in which 19chemosensory-related genes were screened, including 7 OBPs, 3 CSPs, 6 ORs, 2 IRs, and 1SNMP (see Tables 1–5). These 19 DEGs were discovered in all eight libraries. To show the expression patterns of these DEGs at each developmental stage, a heatmap was constructed on the basis of the RPKM values (Fig 2). Based on the heatmap, the correlation between biological replicates was well established, and all the correlation coefficients exceeded 0.9 (*P*<0.05), supporting the reliability of the RNA-Seq data. We were surprised to discover that the expression levels of the 19 chemosensory genes changed significantly at the T1 period(for both T1-1 and T1-2) compared to those of the other three periods, and all the DEGs were generated from T1.This may be attributable to the fact that the newly emerged bees have undergone great changes as a consequence of physical or environmental factors, or that they may be more sensitive to odorant molecules than the adults. On the one hand, among the 19 DEGs, 6 were downregulated (including all 3 CSPs, 2 OBPs and 1 IR), in which *OBP13*, *OBP17*, *CSP5*, and *CSP6*showed great changes. The remaining13DEGs were upregulated (including all 6 ORs, 5 OBPs, 1 IR, and 1 SNMP), in which *OBP7* and *OBP15*changed greatly. Presumably, these downregulated olfactory genes were related to the stimulatory influence of environmental variation, while the upregulated genes were connected with working behavior.

We speculate that there may be synergistic effects among the up- or down-regulated genes, but the specific mechanism still requires further in-depth analysis. In contrast to T1, the level of expression in the other three developmental periods did not vary significantly. In other words, expression of all olfactory-related genes in adult bees remained relatively stable. These data imply that one olfactory gene may act on specific odor molecules; this ability was relatively stable in adult bees and did not change dramatically with the variation of individual behavior and the outer environment.

**Fig 3:**
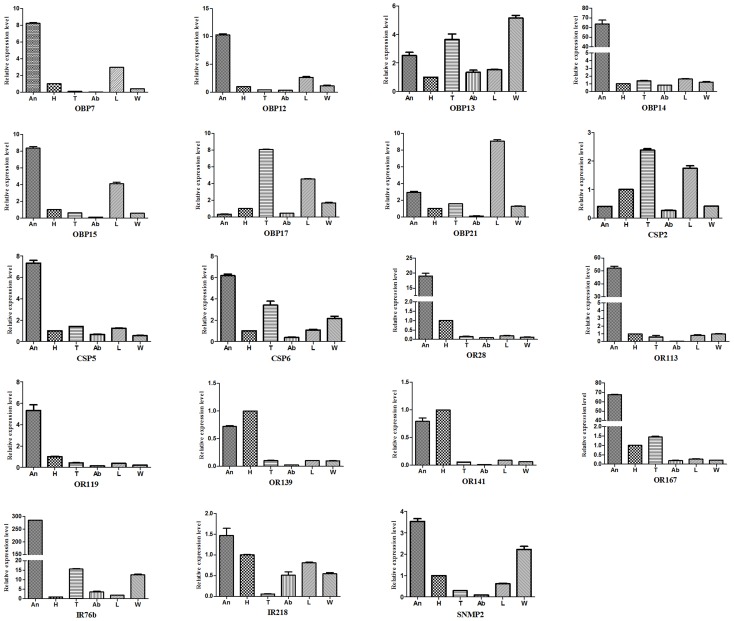
Tissue distribution of 19 putative chemosensory DEGs using qPCR. The X-axis shows different tissues. The Y-axis shows the relative mRNA expression level. An, antennae; H, head; T, thorax; Ab, abdomen; L, legs; W, wings. The standard error is represented by the error bars above the columns.

Nine DEGs were randomly chosen for the purpose of validating the results of the Solexa sequencing using qPCR. The qPCR results showed that the expression pattern of 8 ofthe9 selected genes was consistent with that of the RNA-Seq analysis (S6 Fig);these results demonstrated that the quality of Solexa sequencing was reliable. In addition, we used qPCR in order to obtain more expression information about the19 DEGs in different tissues. Five genes (*OBP14*,*OR28*, *OR113*, *OR167*,and *IR76b*) were highly expressed in antennae. Eight genes (*OBP7*, *OBP12*, *OBP15*, *CSP5*, *CSP6*, *OR119*, *IR218*, and *SNMP2*) were relatively enriched in antennae. Two genes (*OR139* and *OR141*) were highly expressed in both antennae and head. Other two genes (*OBP17*and *CSP2*) showed higher expression in thorax. *OBP21* was expressed more in legs, and *OBP13* was expressed more in wings (Fig 3). An interesting feature of the expression profiles was that when a gene was highly expressed in the thorax, it was also highly in legs, but was weakly in antennae.

The expression profiles of OBPs and CSPs involved in sex, tissue and age in the honeybee have been investigated in previous studies. Due to the different materials selected in different studies, it is hard to perform a detailed comparison among the different studies. Here we compared the tissue-related expression patterns of OBPs and CSPs between this study and previous reports. The expression patterns of *OBP7*, *OBP13*, *OBP15* and*OBP17* were similar to the data of Forêt and Maleszka [26]. However, some results showed inconsistencies; for example, *OBP12* was identified in the antennae in this study, but was only detected in the queen ovaries of *A*. *mellifera* [26]. This suggests that *OBP12* may play a different physiological role in the two species. With respect to CSPs, *CSP5* and *CSP6* were reported to be relatively highly expressed in antennae [34], in agreement with our data. This result indicates that *CSP5* and *CSP6* may be involved in chemical sensory activities. Additionally, there were evident differencesin*CSP2* between this study and that of Li and coauthors [34], while our data was similar with the expression pattern in *A*. *mellifera* [27].

Our analyses revealed two main categories of expression patterns within these genes. One pattern showed that most DEGs were abundantly expressed in the antennae; again verifying that the antennae are the major olfactory organ of insects [49]. Other patterns revealed that some genes were enriched in other non-chemosensory organs, such as the thorax, legs, and wings. This expression pattern indicates that some chemosensory sensillae are located on other body parts, such as on the thorax stegma, the leg tarsi, and the wing margin, or that these genes participate in physiological processes other than chemoreception [50–52].

In addition, we found that all OR DEGs were 5 to 50 times more enriched in the antennae, compared with the thorax, abdomen, legs, and wings, which was similar to the expression pattern of other hymenopteran ORs, such as *A*. *mellifera* [29], *Cotesia vestalis* [53], and *Microplitis mediator* [54]. Our expression profiling results suggest that the ORs of insects are highly restricted in the antennae and may play important and special role in olfactory recognition.

## Conclusion

In this study, we not only identified 109 putative chemosensory genes of *A*. *c*. *cerana*, acquiring19 DEGs for different developmental stages by antennal transcriptome analysis, but also investigated expression profiles of these DEGs. As a result, we obtained worthy information. This research serves as a valuable resource in the identification of characteristics on the olfactory recognition mechanism of *A*. *c*. *cerana*, making it possible for further research on functional analyses of these genes.

## Supporting Information

S1 Fig Phylogenetic tree of candidate AcerOPBs with other hymenopteran OBPs. Acep, *Atta cephalotes*; Acer, *Apis cerana*; Amel, *Apis mellifera*; Cflo, *Camponotus floridanus*; Evir, *Euglossini viridissima*; Mmed, *Microplitis mediator*. The clade in blue indicates the Minus-C class of *A*. *c*. *cerana* and *A*. *mellifera*.
(TIF)

S2 Fig Phylogenetic tree of candidate AcerCSPs with other hymenopteran CSPs. Acep, *Atta cephalotes*; Acer, *Apis cerana*; Amel, *Apis mellifera*; Cflo, *Camponotus floridanus*; Evir, *Euglossini viridissima*.
(TIF)

S3 Fig Phylogenetic tree of candidate AcerORs with other hymenopteran ORs. Acer, *Apis cerana*; Amel, *Apis mellifera*; Nvit, *Nasonia vitripennis*. The clade in blue indicates the Orco class.
(PDF)

S4 Fig Phylogenetic tree of candidate AcerIRs with other hymenopteran IRs. Acep, *Atta cephalotes*; Acer, *Apis cerana*; Amel, *Apis mellifera*; Cflo, *Camponotus floridanus*;Nvit, *Nasonia vitripennis*. The clade in blue indicates the IR8a/IR25a class.
(TIF)

S5 Fig Phylogenetic tree of candidate AcerSNMPs with other hymenopteran SNMPs. Acep, *Atta cephalotes*; Acer, *Apis cerana*; Ador, *Apis dorsata*; Aflo, *Apis florea*; Amel, *Apis mellifera*; Bter, *Bombus terrestris*; Hlab, *Habropoda laboriosa*; Hsal, *Harpegnathos saltator*; Pcan, *Polistes canadensis*.
(TIF)

S6 Fig Comparison diagrams between the RPKM values and the qPCR results of 9 DEGs. (TIF)

S1 Table Primers used for qRT-PCR analysis on DEGs. (DOC)

S2 Table Evaluation statistical table of high quality sequencing data. (DOCX)

S3 Table Distribution of unigene size in the transcriptome assembly. (DOCX)

S4 Table Correlation statistics between biological replicate samples. (DOCX)
