# Supplementary figures and images for: Antennal Transcriptome and Differential Expression Analysis of Five Chemosensory Gene Families from the Asian Honeybee Apis cerana cerana
Source: PLoS One. 2016 Oct 24;11(10):e0165374. doi: 10.1371/journal.pone.0165374 (PMC5077084; doi:10.1371/journal.pone.0165374)

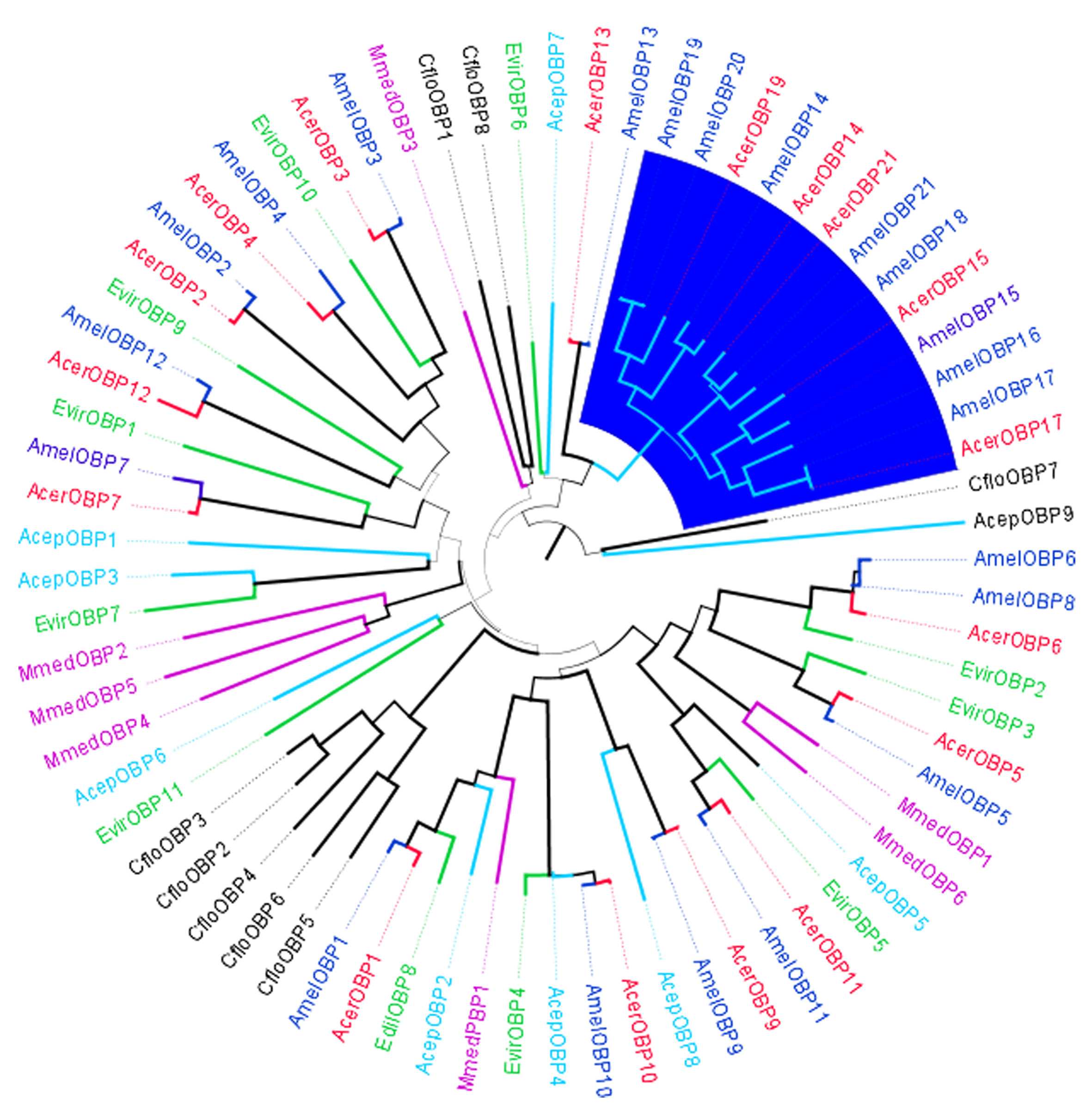

Supplement: S1 Fig — Acep, Atta cephalotes; Acer, Apis cerana; Amel, Apis mellifera; Cflo, Camponotus floridanus; Evir, Euglossini viridissima; Mmed, Microplitis mediator. The clade in blue indicates the Minus-C class of A. c. cerana and A. mellifera. (TIF) [file pone.0165374.s001.tif]

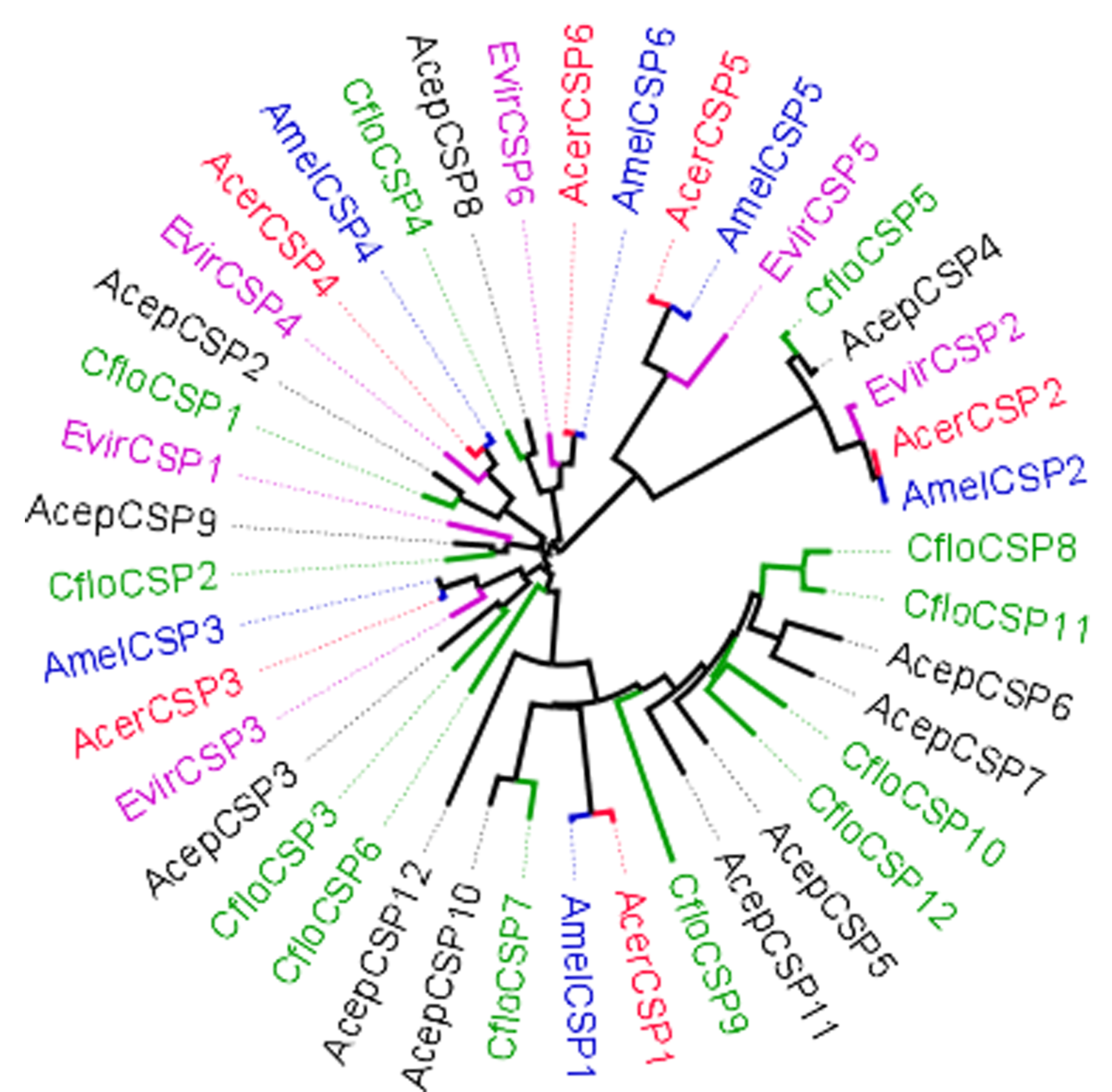

Supplement: S2 Fig — Acep, Atta cephalotes; Acer, Apis cerana; Amel, Apis mellifera; Cflo, Camponotus floridanus; Evir, Euglossini viridissima. (TIF) [file pone.0165374.s002.tif]

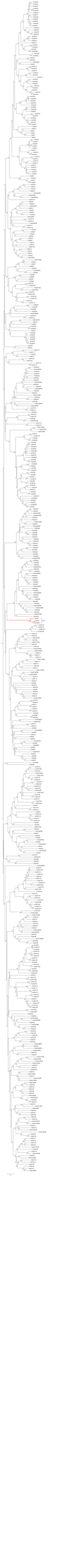

Supplement: S3 Fig — Acer, Apis cerana; Amel, Apis mellifera; Nvit, Nasonia vitripennis. The clade in blue indicates the Orco class. (PDF) [file pone.0165374.s003.pdf]

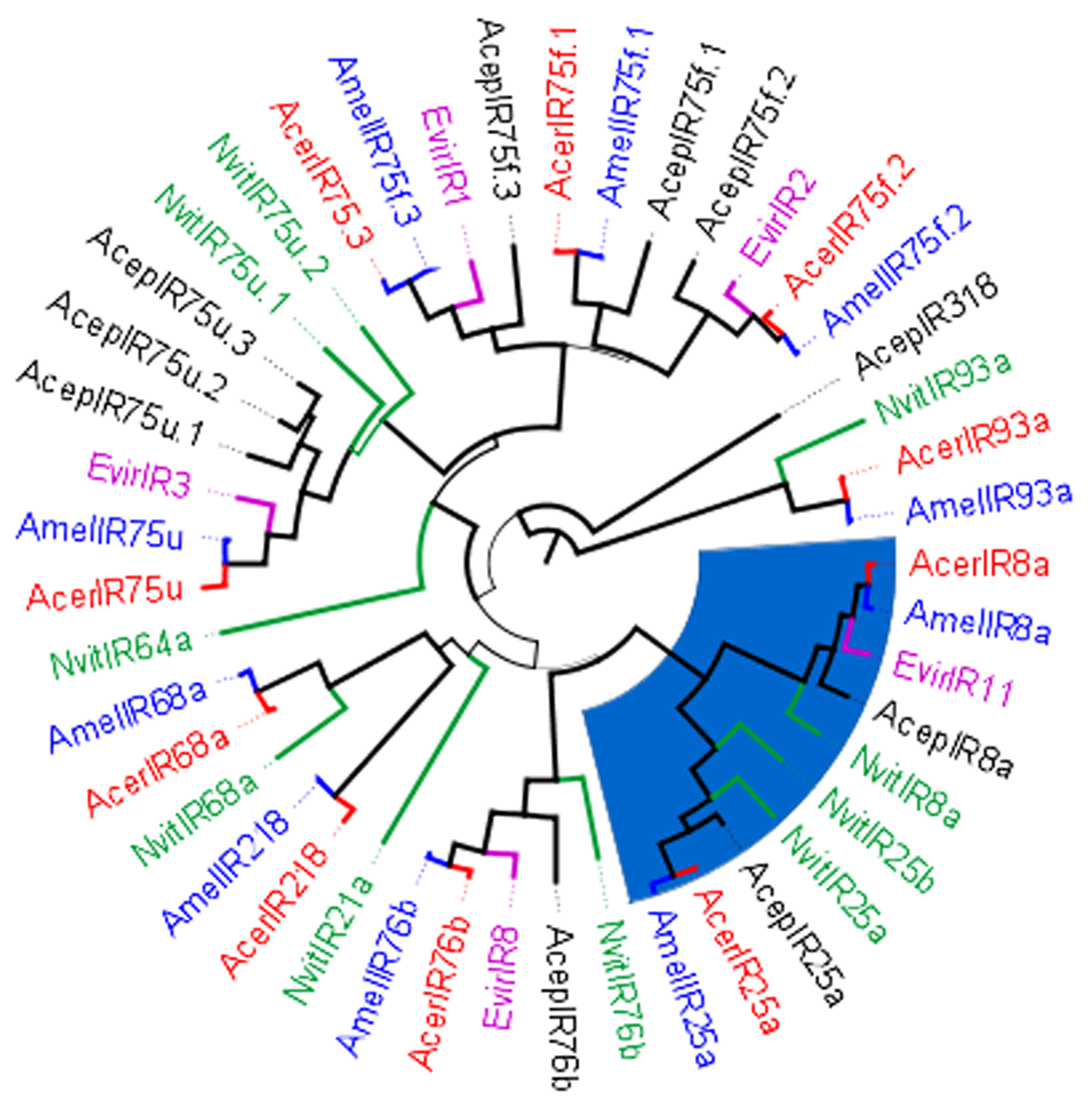

Supplement: S4 Fig — Acep, Atta cephalotes; Acer, Apis cerana; Amel, Apis mellifera; Cflo, Camponotus floridanus;Nvit, Nasonia vitripennis. The clade in blue indicates the IR8a/IR25a class. (TIF) [file pone.0165374.s004.tif]

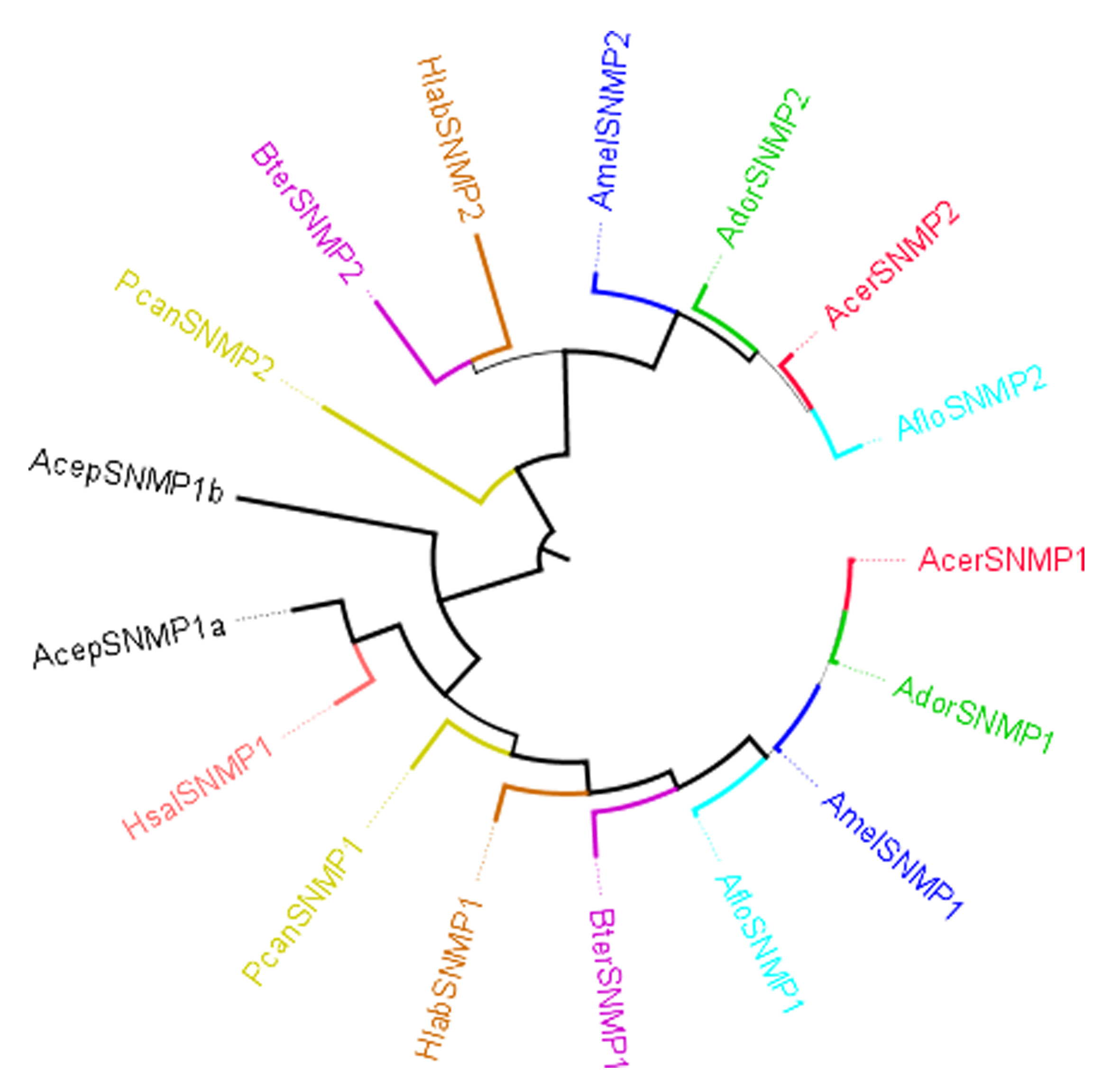

Supplement: S5 Fig — Acep, Atta cephalotes; Acer, Apis cerana; Ador, Apis dorsata; Aflo, Apis florea; Amel, Apis mellifera; Bter, Bombus terrestris; Hlab, Habropoda laboriosa; Hsal, Harpegnathos saltator; Pcan, Polistes canadensis. (TIF) [file pone.0165374.s005.tif]

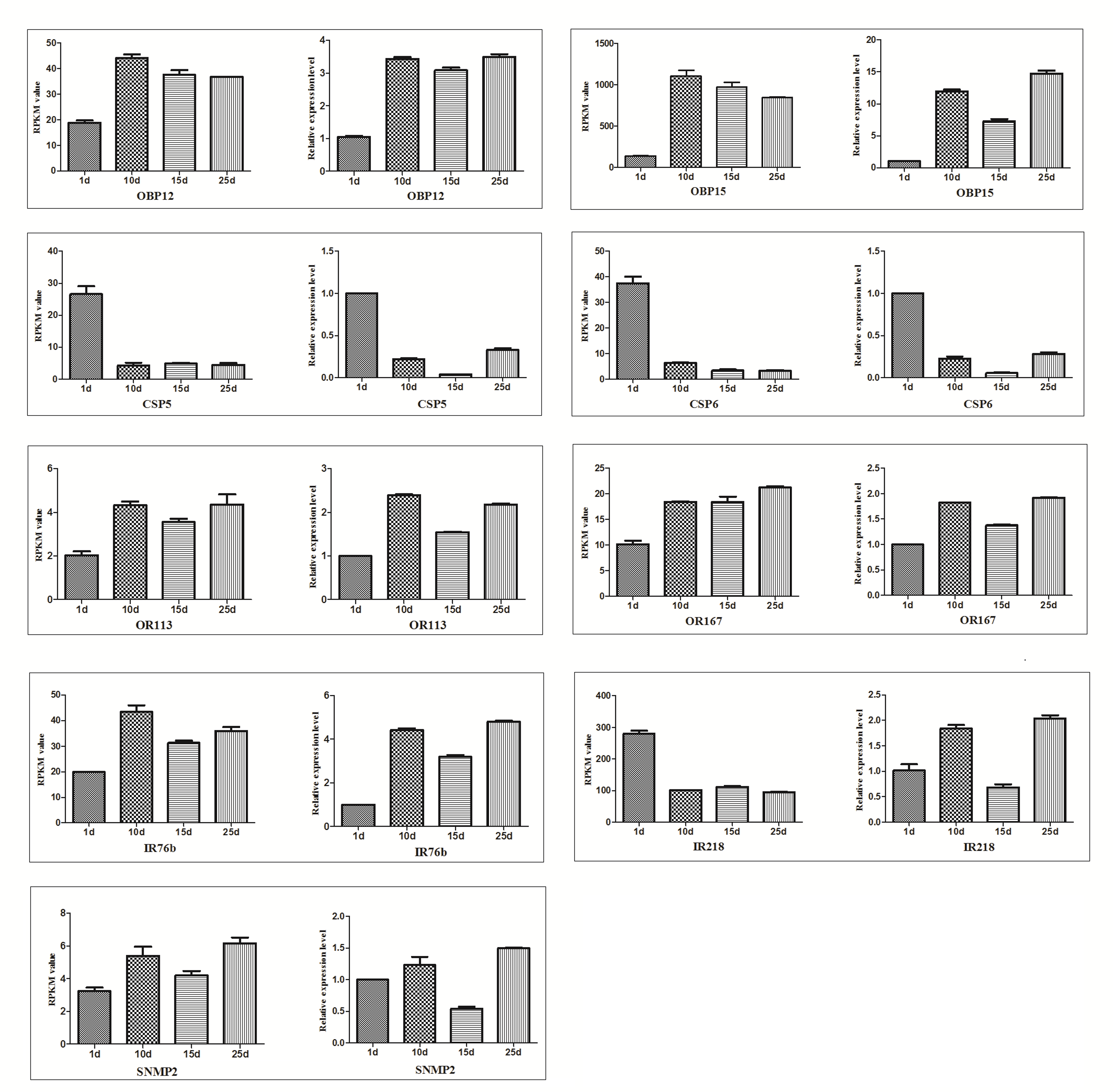

Supplement: S6 Fig — (TIF) [file pone.0165374.s006.tif]
