# Supplementary material for: Antennal Transcriptome and Differential Expression Analysis of Five Chemosensory Gene Families from the Asian Honeybee Apis cerana cerana
Source: PLoS One. 2016 Oct 24;11(10):e0165374. doi: 10.1371/journal.pone.0165374 (PMC5077084; doi:10.1371/journal.pone.0165374)
Supplement: S1 Table — (DOC) [file pone.0165374.s007.DOC]

**S1Table. Primers used for qRT-PCR analysis on DEGs.**

| Primer name | Forward primer (5**'**-3**'**) | Reverse primer (5**'**-3**'**) |
| --- | --- | --- |
| Odorant binding proteins (OBPs) | | |
| OBP7 | CTTTCCGTTGCCGTAATCAT | TTCCTCCGATATGTCTTCCTCT |
| OBP12 | ATGAATGGCTCCGAATTGAG | GCGACGTCACACTTGTCATT |
| OBP13 | AGCAGACGACGTTAAGAAGGG | CGTTGAAAGTTGTGTCTGCGT |
| OBP14 | GGCTTTTGCATTTGCGTTGG | CAATGCCAGTTTCTGTGGCG |
| OBP15 | TGCTATTTGGATTTGCGTTG | AGTTTGTGCGCTACACATCG |
| OBP17 | TGCTATTTGCGTTTGCGTTA | CGTCGTCCATATTGATCTTGC |
| OBP21 | ATGAAATTCGTTATTTTCAGTT | TCTTAGGGTCATCGTGCT |
| Chemosensory proteins (CSPs) | | |
| CSP2 | GGCAGAAACGGAAGAAGGA | CTCAAAACCAGTGGTGCTAAAC |
| CSP5 | TTTTGGATCGGGGACATTG | CAACTGCCACTCATAGGGATAAT |
| CSP6 | GCAGAATGGTCGTATCCTCAC | TACCTTCGTTAGTACATGGTCCTT |
| Odorant receptors (ORs) | | |
| OR28 | GGCAAACAGTAACAAGGGAAG | CAAAATAGCCGCCGAAATAG |
| OR113 | CGTTACGATGGACTATTGTTTG | TCATTGCACGAATCTATCACG |
| OR119 | CTTGATCACGATGCTGTTGG | ATGATCGAGGTGCTGGAAAG |
| OR139 | CGAAACTTGTGGAGCTTTATCG | GGCACCATAAACTGTATTCCTG |
| OR141 | TATTGTTTCGTGCGGAGATG | TATATCGTGCGGTGACCACCT |
| OR167 | GCGTAAGCACTACTTTGCCTA | CAGCGAAAGCATTAGTCCAA |
| Ionotropic receptors (IRs) | | |
| IR76b | GCCGATGTTTACTCTGCCTTCT | GTTTCCTTCATTTGTCGCCTTT |
| IR218 | ATGGGTTCGAAGCTGAAGTG | AACGCGATATCCACCTTTTG |
| Sensory neuron membrane proteins (SNMPs) | | |
| SNMP2 | AAGAAGGCGAAAAGGACGG | AAGGCAATGGATTTACTAATGGTG |
| Housekeeping gene | | |
| Arp1 | ACTACGGCCGAACGTGAAAT | GGAAAAGAGCCTCGGGACAA |
