# Supplementary material for: Antennal Transcriptome and Differential Expression Analysis of Five Chemosensory Gene Families from the Asian Honeybee Apis cerana cerana
Source: PLoS One. 2016 Oct 24;11(10):e0165374. doi: 10.1371/journal.pone.0165374 (PMC5077084; doi:10.1371/journal.pone.0165374)
Supplement: S2 Table — (DOCX) [file pone.0165374.s008.docx]

| Samples | Total reads | Total bases | GC (%) | Q30% |
| --- | --- | --- | --- | --- |
| T1-1 | 22646116 | 4573559618 | 41.88 | 94.21 |
| T1-2 | 19729880 | 3984574328 | 41.48 | 94.53 |
| T2-1 | 18572570 | 3750951188 | 41.64 | 94.26 |
| T2-2 | 16680304 | 3368432785 | 43.35 | 94.24 |
| T3-1 | 15978265 | 3226884637 | 41.73 | 95.05 |
| T3-2 | 19480786 | 3934262219 | 41.26 | 95.19 |
| T4-1 | 18227789 | 3681240373 | 42.09 | 94.97 |
| T4-2 | 17054413 | 3444348276 | 42.11 | 94.89 |

**S2 Table. Evaluation statistical table of high quality sequencing data.**

T1-1, T1-2 − biological repetition samples of 1-day-old workers.

T2-1, T2-2 − biological repetition samples of 10-day-old workers.

T3-1, T3-2 − biological repetition samples of 15-day-old workers.

T4-1, T4-2 − biological repetition samples of 25-day-old workers.
