# Supplementary material for: Antennal Transcriptome and Differential Expression Analysis of Five Chemosensory Gene Families from the Asian Honeybee Apis cerana cerana
Source: PLoS One. 2016 Oct 24;11(10):e0165374. doi: 10.1371/journal.pone.0165374 (PMC5077084; doi:10.1371/journal.pone.0165374)
Supplement: S3 Table — (DOCX) [file pone.0165374.s009.docx]

**S3 Table. Distribution of unigene size in the transcriptome assembly.**

| Unigenes Length | Total Number | Percentage |
| --- | --- | --- |
| 200-300 | 35001 | 27.98% |
| 300-500 | 36495 | 29.18% |
| 500-1000 | 30097 | 24.06% |
| 1000-2000 | 14398 | 11.51% |
| 2000+ | 9080 | 7.26% |
| Total number | 125072 |  |
| Total length | 95026575 |  |
| N50 length | 1151 |  |
| Mean length | 759.78 |  |
