# Supplementary material for: Antennal Transcriptome and Differential Expression Analysis of Five Chemosensory Gene Families from the Asian Honeybee Apis cerana cerana
Source: PLoS One. 2016 Oct 24;11(10):e0165374. doi: 10.1371/journal.pone.0165374 (PMC5077084; doi:10.1371/journal.pone.0165374)
Supplement: S4 Table — (DOCX) [file pone.0165374.s010.docx]

**S4 Table. Correlation statistics between samples biological repetition.**

| Sample1 | Sample2 | R^2^ |
| --- | --- | --- |
| T1-1 | T1-2 | 0.9924 |
| T2-1 | T2-2 | 0.9886 |
| T3-1 | T3-2 | 0.9977 |
| T4-1 | T4-2 | 0.9970 |

R^2^ − the squared of Pearson correlation coefficient.

T1-1, T1-2 − biological repetition samples of 1-day-old workers.

T2-1, T2-2 − biological repetition samples of 10-day-old workers.

T3-1, T3-2 − biological repetition samples of 15-day-old workers.

T4-1, T4-2 − biological repetition samples of 25-day-old workers.
